# Editorial: The role of epithelial cell-microbe interactions in oral health and disease

**DOI:** 10.3389/froh.2022.1044369

**Published:** 2022-10-21

**Authors:** O.A. Gonzalez, S.M. Wallet, R.J. Lamont

**Affiliations:** ^1^ Center for Oral Health Research, College of Dentistry, University of Kentucky, Lexington, KY, United States; ^2^ Department of Oral Biology, College of Dentistry, University of Florida, Gainesville, FL, United States; ^3^ Oral Immunology and Infectious Diseases, School of Dentistry, University of Louisville, Louisville, KY, United States

**Keywords:** epithelial, oral bacteria, oral dysbiosis, oral symbiosis, periodontal, oral health, innate immunity

**Editorial on the Research Topic** The role of epithelial cell-microbe interactions in oral health and disease

Mucosal surfaces are continuously exposed to a vast number of organisms with a spectrum of pathogenic potential, which represent a constant immunological challenge. Nevertheless, microbes and host have co-evolved and developed a symbiotic relationship, in which both can co-exist without causing disease in a tolerogenic environment. The cellular and molecular mechanisms associated with immunological tolerance at mucosal surfaces including the oral mucosa, as well as the events related to its disruption, which lead to dysbiosis and inflammatory disease, remain unclear and are thus a current area of intense research. To date, significant attention has been placed on how oral bacteria modulates the immune-inflammatory compartment (i.e., neutrophils, monocytes/macrophages, dendritic cells, and lymphocytes) mainly in the context of disease. Nevertheless, the role of epithelial cell-microbe interactions has been significantly less studied in oral health and disease. It is becoming clear, from both research in the oral and gastrointestinal field, that rather than passive players, epithelial cells are actively interacting with the microbiome as well as with the immune cell compartment, and their role in both maintaining oral health and initiating inflammatory disease is significant.

The goal of this research topic is to ignite a discussion of current work associated with the role of epithelial cell-microbe interactions in oral health and disease. Specifically, we seek to review general evidence and identify potential gaps in research of oral epithelial responses that could help the field to improve the knowledge on this active and crucial -but poorly studied- cellular player of mucosal immunity. This issue is particularly focused on epithelial cells and the oral chronic inflammatory disease of periodontitis.

The ability of specific oral bacterial species to invade and disrupt the epithelial barrier as well as evade the mucosal immune system are both key determinants in the identification of bona fide periodontopathogens. Nevertheless, how pathogens modulate oral epithelial responses that initiate disease remains unclear. Takeuchi et al., presents a mechanism used by the periodontopathogen *Porphyromonas gingivalis*, in disrupting the epithelial barrier by targeting junctional adhesion molecules. For this, the authors used 3-D organotypic gingival tissue models, and showed that gingival epithelial barrier disruption involved the proteolytic activity of the pathogen-produced enzymes gingipains, which allows *P. gingivalis* penetration of epithelial tissues as well as delivery of virulence factors such as lipopolysaccharide and proteases at deeper levels in gingival tissues. These findings along with reports from additional groups support the clear permeation of virulence factors of *P. gingivalis* in gingival tissues obtained from periodontal disease sites in humans as well as their presence in distant places such as blood and brain.

Similarly, the work of da Silva et al., demonstrated that *A. actinomycetemcomitans (Aa)* modulates apoptosis in oral epithelial cells (OECs) and attenuates CXCL8 responses, specifically through one of its outer membrane proteins (OMP29). Although these findings require further validation in preclinical and clinical models, it seems to be an interesting target for future studies aimed at attenuating *Aa* virulence, particularly in more severe/aggressive forms of periodontitis.

Another important pathogen associated with oral inflammatory disease as well as colorectal cancer is *Fusobacterium nucleatum*. Groeger et al., provide a detailed review outlining the cellular and molecular mechanisms through which *F. nucleatum* is able to invade oral epithelial cells as well as enhance inflammation and periodontal damage. A description of the major adhesins of this oral pathogen as well as the host receptors and cell signaling pathways identified as targets for these adhesins, is presented. The identification and elucidation of the molecular pathways used by *F. nucleatum* to disrupt homeostasis and cause disease would open new possibilities to prevent or attenuate both the oral and systemic pathologic effects.

Part of the homeostatic interactions between bacteria and oral epithelium in health involves a precisely orchestrated expression of antimicrobial proteins that allows higher abundance of commensal bacteria with lower levels of pathobionts as well as modulation of bacterial virulence. Variations in the expression of antimicrobial proteins induced by environmental, host factors, or pathogens could lead to significant changes in the abundance of bacterial species favoring growth of pathogens (i.e., dysbiosis) as well as increase the likelihood for invasion and persistent infection of epithelial surfaces. Johnstone and Herzberg in a comprehensive review highlighted the crucial role of antimicrobial responses derived from the epithelial barrier with particular emphasis on defensins and calprotectin. Improved knowledge about what specific oral epithelial antimicrobial protein/peptide responses are critical for health and what particular variations in those responses can explain oral dysbiosis and early events of periodontitis is clearly needed.

Finally, using the ligature-induced periodontitis nonhuman primate model (NHP) combined with transcriptomics, Gonzalez et al.'*s* group evaluated ∼450 genes related to epithelial cell structure and function during different phases of disease. Although an extensive alteration in the epithelial cell functions occurred rapidly after initiation of disease and persisted throughout the progression, clinical resolution of disease was not always consistent with complete biological re-establishment of basal transcriptome epithelial responses, which may imply persistent but clinically undetectable risk for recurrence of tissue damage/disease. This reinforces the importance of continuing to work on integrating the oral molecular profiles or signatures developed through omics technologies with the clinical activities that could allow the identification of subjects more susceptible or resistant to disease progression, or patients with biological failure to properly resolve disease. The use of NHP, which demonstrates clinical, microbiological, and immunological features of human disease, will be helpful in the process of identifying such molecular signatures, particularly related to specific and well-demarcated phases of disease that cannot be derived from human studies.

Future issues dedicated to the role of epithelial-bacteria interactions in oral health are warranted. A better understanding of how epithelial responses to a symbiotic oral microbiome actively regulate pathologic inflammatory disease is of paramount importance to dissect the very early cellular and molecular events of oral disease that occur even in the absence of frank tissue damage/destruction. Such specific and early epithelial responses in health could be used towards the development of future preventive strategies based on early detection of dysbiosis/disease and host modulation strategies.

